# Phytotherapy for Cachexia: Where Do We Stand?

**DOI:** 10.3389/fphar.2020.00917

**Published:** 2020-07-08

**Authors:** Kenny Kuchta, Silke Cameron

**Affiliations:** Clinic for Gastroenterology and Gastrointestinal Oncology, University of Göttingen Medical School, Göttingen, Germany

**Keywords:** phytotherapy, cachexia, Kampo (traditional Japanese herbal medicine), Hozai, adaptogen, mechanistic target of rapamycin, melatonin, interleukin

## Abstract

**Background:**

In contrast to Western medicine which currently offers no approved pharmacotherapy options for cachexia, in Japan multi-component extracts of medicinal plants are used with coverage by the national health insurance. This so called “Kampo” medicine is an example of the modern concept of multi-component/multi-target therapy. For the three traditional preparations Hochuekkito (補中益気湯), Juzentaihoto (十全大補湯), and Rikkunshito (六君子湯), a multitude of clinical research data relating to cachexia has been published. These preparations are also referred to as “Hozai” (補剤). A similar concept is found in Russian herbal medicine, where the term “Adaptogen” was coined for pharmacologically active substances which enhance adaptive stress repose.

**Methods:**

Scientific literature—including original Japanese articles—was reviewed regarding the effects of these herbal preparations on cachexia. Cachexia is a complex set of symptoms including muscle atrophy with loss of weight, fatigue, and weakness.

**Results:**

In a 1985 study by Kuroda et al., Hochuekkito showed efficacy in involuntary weight loss and fatigue in 63% of 162 patients with genitourinary cancer. For cancer-related fatigue, a significant improvement was reported within 2 weeks by Jeong et al. in 2010. In patients with chronic fatigue syndrome, Hochuekkito showed an overall improvement with 8–12 weeks of therapy in a 1997 study by Kuratsune et al. In a 2005 randomized placebo-controlled trial by Satoh et al. on 13 geriatric Q1 patients in a 16-week treatment protocol, Hochuekkito showed significant improvement of general health, physical functioning and the Profile of Mood States (POMS). In 71 geriatric COPD patients in a 2009 placebo-controlled randomized study, Tatsumi et al. found a significant body weight increase and a CRP, TNF-α, IL-6 decrease over 6 months of therapy. For Juzentaihoto in 48 hepatocellular carcinoma patients, Tsuchiya et al. 2008 documented a significantly longer recurrence-free survival (49 vs. 24 months) as compared to the control group (p=0.023). For the much simpler Rikkunshito prescription, a 2011 retrospective study by Fujitsuka et al. on 39 Stage III/IV pancreatic cancer patients treated with Gemcitabine (n=33) or Gemcitabine/Rikkunshito (n=6) showed a significantly prolonged median survival with 224 vs. 378.5 days (p < 0.05). In a 2011 open-label clinical study by Utumi et al. on geriatric cachexia in 6 dementia patients, treatment with Rikkunshito for 4 weeks resulted in a significant body weight increase. In all the above studies, the standardized dosage of 3x2.5 g/d extract granules for most Japanese health insurance-covered Kampo extract-preparations was applied.

**Conclusion:**

As there is currently no accepted pharmacotherapy option for cachexia available in the West, a transfer of these East Asian gold standard prescriptions into the European market would be desirable. We were further able to demonstrate that the mTOR, interleucin, and melatonin pathways are modified by herbal compounds which thus counteract cachexia. More research in this field is urgently needed in order to provide new, effective treatments for cachexia patients.

## Introduction

Cachexia is commonly defined as a devastating state of involuntary weight loss that occurs as a complication in the final stage of numerous cancers, as well as infectious and inflammatory diseases, contributing in all cases to a significantly increased mortality (“Cachexia.”; Inui, 1999). Systemic inflammation from these conditions can cause detrimental changes to metabolism and body composition (von Haehling et al., 2017). Western medicine currently offers no approved pharmacotherapy options for cachexia, which cannot be reversed by nutritional supplementation.

In traditional herbal medicine, these wasting syndromes—irrespective of the formerly unknown underling diseases—have been treated with herbal extracts or their combinations. In East Asia, mainly combination prescriptions are used, containing drugs such as *Panax ginseng* C.A.Mey., *Wolfiporia extensa* (Peck) Ginns (1984) (Syn. *Poria cocos*), *Ziziphus jujuba* Mill., and *Glycyrrhiza glabra* L. Commonly used prescriptions for this indication are referred to as “Hozai” (補剤). For the Hozai prescriptions Hochuekkito (補中益気湯), Juzentaihoto (十全大補湯), and Rikkunshito (六君子湯) a multitude of clinical research data relating to cachexia has been published, resulting in individual monographs in (JPXVII, 2016).

In Russian traditional herbal medicine mostly single herbs were used, such as *Aralia mandshurica* Rupr. et Maxim., *Echinopanax elatum* Nakai, *Eleutherococcus senticosus* (Rupr. & Maxim.) Maxim., *Leuzea carthamoides* (Willd.) DC., *P. ginseng* C.A.Mey., *Schisandra chinensis* (Turcz.) Baill., *Rhodiola rosea* L., *Sterculia platanifolia* L.f. These are also referred to as “Adaptogens”. This term was defined by Nicolai V. Lazarev as medicinal substances that increase the resistance of the organism to non-specific external and internal stressors (Lazarev, 1958).

## Pharmacological Targets Against Cachexia

In recent years, research has uncovered several connections between the underlying regulatory pathways of the cachexia syndrome and the molecular pharmacological mechanisms of actions of herbal drugs traditionally used in this indication.

For instance, the mechanistic target of rapamycin (mTOR), a major intracellular signaling intermediary, participates in cell growth by up-regulating anabolic processes such as protein and lipid synthesis (Yoon, 2017) thus counteracting cachexia. It has further been shown that mTORC1 is decreased throughout cachexia in gastrocnemius muscle of ApcMin/+ mice, a model of colorectal cancer (White et al., 2011) as well as other tumor models (Puppa et al., 2012; White et al., 2013; Puppa et al., 2014). In this regard, bioactive constituents of i.e. Ginseng activate the PI3K/AKT/mTOR pathways in a model of liver regeneration after surgery in mice (Zhong et al., 2019). It has further been shown, that components of Ginseng induce autophagy by regulating the same pathway in a tumor model of esophageal cancer (Liu et al., 2019). Also, in the case of the common adaptogen *Rhodiola rosea* L., effects on the mTOR pathway could be experimentally demonstrated, as its major active constituent salidroside was shown to alleviate cachexia symptoms in mouse models of cancer cachexia *via* activating mTOR signaling (Chen et al., 2016).

A further mechanism of cachexia is increased inflammation. Major mediators of inflammation are pro-inflammatory cytokines such as interleukin-6 (IL-6), which activates the STAT pathway. Prolonged activation of the IL6/JAK/STAT axis is an established mechanism not only in tumorigenesis but also in muscle wasting in cancer cachexia (Narsale and Carson, 2014). Ginseng saponins have been shown to reduce plasma levels of IL-6 in a mouse stress model (Kim et al., 2003). Furthermore, the levels of IL-6 and Interferon-1β (IFN-1β) were shown to decrease after administration of Ginseng saponis in a model of aging mice (Yu and Li, 2000). It has been shown, that ginsenoids further ameliorate intestinal function by suppressing intestinal inflammation and promoting intestinal barrier repair (Dong et al., 2019). Isoliquiritigenin, a flavonoid compound of licorice, has also been shown to block M2 macrophage polarization in colitis-associated tumorigenesis through down regulating i.e. IL-6 (Zhao et al., 2014). Similarly, constituents of *Rhodiola rosea* decrease the production of TNF-α, Il-1β, IL-6 as well as iNOS in a model of lipopolysaccharide (LPS) induced inflammation in microglia cells (Lee et al., 2013).

A third pathway is melatonin (Panossian et al., 2018). Melatonin not only regulates the sleep wake cycle and is relevant in neurohormonal signaling involving glucocorticoid and catecholamine-related stress response, but is also involved in catabolic mechanisms inducing muscle wasting in cachexia (Jafari-Vayghan et al., 2019). In this context, Ginseng saponins induce expression of melatonin receptor mRNA in a rat model gastric stress (Deng et al., 2008). As for Glyzyrrhizae radix, it has been shown that melatonin itself is secreted by the roots (Afreen et al., 2006). As for *Rhodiola rosea* it was demonstrated that salidroside induces melatonin (urinary excretion) in mice (Ma et al., 2015). Hence, the mTOR, interleukin and melatonin pathways form essential components of both cachexia and the adaptogenic effect.

**Figure 1:**
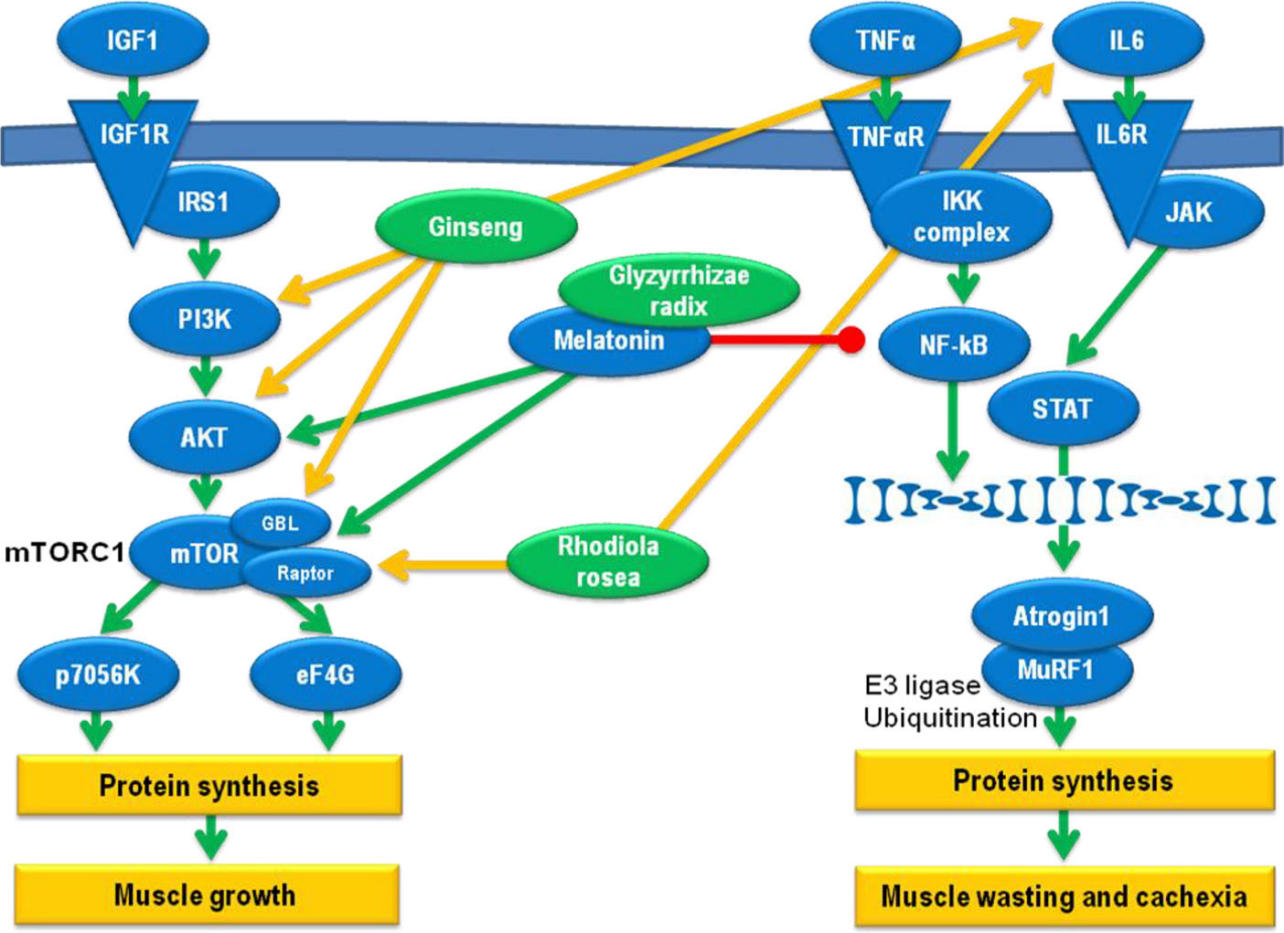
Pathways relevant for the adaptogenic effect against cachexia development. Green arrows: induction, red dash: inhibition, yellow arrows: effect of herbal extracts.

An overview of the different adaptogenic pathways pertaining to cachexia is given in **Figure 1**.

## Clinical Approaches to Cachexia Treatment

In conventional medicine, there are currently no approved therapies for involuntary weight loss such as in cachexia (von Haehling et al., 2009). In the case of cachexia in cardiac disease, angiotensin-converting enzyme (ACE) inhibitors and beta-blockers have been recommended (von Haehling et al., 2009) but these mainly treat the underlying heart disease and not cachexia itself. Due to the close connection of cachexia with pro-inflammatory processes in the body (von Haehling and Anker, 2005), cachexia patients should be treated with a high caloric diet consisting of foods that counteract inflammatory responses such as fish oil, olives, walnuts, flaxseed oil, any fruits or vegetables, garlic, ginger, turmeric, sunflower seeds, eggs, herring, or nuts (Azhar and Wei, 2006). For the same reasons, pharmacotherapy with inhibitors of pro-inflammatory cytokines has been proposed as a therapy option for cachexia (von Haehling et al., 2009) and numerous anti-inflammatory agents like tumor necrosis factor-α inhibitory antibodies, lipopolysaccharide inhibitors, proteasome inhibitors, or the classical statins have been researched (von Haehling et al., 2009). If possible, physical activity should also be realized. However, as a final verdict on all these approaches one can state that “therapies that targeted specific single cytokines have largely failed, and it appears that broader approaches are required” (von Haehling et al., 2009).

Unbeknownst to Western practitioners, such broader approaches have already been implemented in Japan, where in the framework of Japanese Kampo medicine multi-component extracts of medicinal plants are used in the treatment of cachexia patients with coverage by the national health insurance (Okumi and Koyama, 2014). Most Kampo preparations consist of extracts of up to 10 medicinal plant drugs, each of which contains hundreds of potentially active substances. This concept of multi-component/multi-target therapy aligns well with current research results in molecular biology, according to which optimal treatment outcomes can only be achieved with a form of therapy that acts simultaneously against the majority of those physiological factors that contribute to the illness. It stands to reason that the efficacy of traditional prescriptions of standardized herbal extract mixtures can therefore be rationally explained based on the assumption that a complex multifactorial pathophysiology (multicausality) can be managed more effectively through the use of a correspondingly composed multi-component mixture than with a single active substance (Wagner, 2006).

Among the 148 insurance covered Kampo extract preparations, traditional prescriptions in the “Hozai” category have proven especially suitable for cachexia therapy (**Table 1**). The term “Hozai” (補剤) translates as “support preparation” and is used to describe preparations that are applied to stop or partially reverse symptoms of physical weakness and degenerative diseases. The two most important single prescriptions of this category are Juzentaihoto (十全大補湯) (Juzentaihoto, 2017) and Hochuekkito (補中益気湯) (Suppl II JP, 2014; Hochuekkito, 2017). They are regarded as effective by the Japanese regulatory authorities and are available as extract preparations of equal quality to European traditional herbal medicinal products.

**Table 1:** Raw drugs and their respective daily dosages in Kampo prescriptions commonly prescribed against cachexia in Japan.

十全大補湯 Juzentaihoto		補中益気湯 Hochuekkito		六君子湯 Rikkunshito	
JP Astragalus Root	3.0 g	JP Astragalus Root	4.0 g	JP Atractylodes Lancea Rhizome	4.0 g
JP Cinnamon Bark	3.0 g	JP Atractylodes Lancea Rhizome	4.0 g	JP Ginseng	4.0 g
JP Rehmannia Root	3.0 g	JP Ginseng	4.0 g	JP Pinellia Tuber	4.0 g
JP Peony Root	3.0 g	JP Japanese Angelica Root	3.0 g	JP Poria Sclerotium	4.0 g
JP Cnidium Rhizome	3.0 g	JP Bupleurum Root	2.0 g	JP Jujube	2.0 g
JP Atractylodes Lancea Rhizome	3.0 g	JP Jujube	2.0 g	JP Citrus Unshiu Peel	2.0 g
JP Japanese Angelica Root	3.0 g	JP Citrus Unshiu Peel	2.0 g	JP Glycyrrhiza	1.0 g
JP Ginseng	3.0 g	JP Glycyrrhiza	1.5 g	JP Ginger	0.5 g
JP Poria Sclerotium	3.0 g	JP Cimicifuga Rhizome	1.0 g		
JP Glycyrrhiza	1.5 g	JP Ginger	0.5 g		

All three preparations and the respective herbal raw drugs that they consist of are monographed in the current version of the Japanese Pharmacopoeia (JP XVII, 2016) (JP, The Japanese Pharmacopoeia).

Hochuekkito has been especially well documented in clinical research to be effective in the treatment of cachexia. In a clinical study on 162 patients suffering from cachexia (i.e. anorexia and fatigue) due to genitourinary cancer, Kuroda et al. (1985) demonstrated its clinical effects on cachexia and further documented improvements in the quality of live and immunological status of these weak, postoperative patients. With a daily dosage of 7.5 g Hochuekkito extract preparation, the efficacy rate was 63.0%, with a rate of effectiveness on anorexia of 48.4% and on lassitude of 36.6%. Although side effects were observed in 7.4% of the patients, these were almost exclusively limited to mild gastrointestinal disorders and no severe adverse effects were observed. The study by Kuroda et al. (1985) therefore indicates that Hochuekkito had clinical effects on cachexia in genitourinary cancer patients.

In a second study, Hochuekkito was found to be effective in the treatment of chronic fatigue syndrome (Kuratsune et al., 1997). In this study, 29 cases of Chronic Fatigue Syndrome (CFS) with a Performance Status (PS) of 2 or more - according to the preliminary CFS diagnostic standard of the Japanese ministry of health - were included. Hochuekkito was administered for 8 weeks to 12 weeks. The degree of tiredness and fatigue as well as the PS value before and after the treatment were examined using an interview table. Immunological parameters were also measured. It is important to note that in this study Hochuekkito was administered in accordance with its Western Medicine indications and not according to traditional Kampo diagnosis. The Performance Status (PS) was systematically evaluated after administration of Hochuekkito and found to be improved in 12 of 29 cases (41.4%). It is of course necessary to take the placebo effect into account when judging the efficacy of medical drugs in a clinical trial, which should be performed as a double-blind trial if possible, which was not the case in Kuratsune et al. (1997). However, the authors point out that the contribution of the placebo effect to the overall efficacy of drugs for the treatment of CFS is typically about 20% as far as PS evaluation is concerned. With an efficacy of 41.4% the observed effect of Hochuekkito was about twice as strong and can therefore not be explained *via* the placebo effect alone (Kuratsune et al., 1997).

In Korea, where this prescription is known under the name “Bojungikki-Tang”, a third randomized clinical trial with Hochuekkito was performed (Jeong et al., 2010). Here, 40 cancer patients with disease related fatigue were randomized into either an experimental group or a control group (waiting list). Whereas patients in the waiting list group did not receive any intervention for 2 weeks, those in the experimental group were treated with Hochuekkito. In the experimental group, statistically significant improvements were documented for the Visual Analogue Scale of Global Fatigue (VAS-F) measuring the severity of fatigue (experimental vs control: −1.1 ± 2.1 vs 0.1 ± 0.9, P < .05) as well as for the Functional Assessment of Cancer Therapy–General (FACT-G), Functional Assessment of Cancer Therapy–Fatigue (FACT-F), and Trial Outcome Index–Fatigue (TOI-F) also showed significant improvements (FACT-G, 3.7 ± 9.9 vs -2.4 ± 9.5, P < .05; FACT-F, 8.0 ± 13.6 vs -2.2 ± 14.1, P < .05; TOI-F, 6.5 ± 9.2 vs -0.5 ± 10.9, P < .05).

As far as its concrete mechanism of action is concerned, Hochuekkito seems to act *via* the activation of the immune system through significantly increasing lymphocyte cell-surface antigens, CD3-positive cells, and CD3/CD4 double-positive cells, as could be demonstrated in a fourth clinical trial on the therapy of chronic weakness in elderly patients (Satoh et al., 2005). Furthermore, clinical research has demonstrated that Hochuekkito acts against chronic fatigue by inhibiting TNF-α, IL-6, IL-10, TGF-1, and INF-production in patients with chronic fatigue syndrome (Shin et al., 2003). It also improves systemic inflammation and nutritional status associated with chronic diseases (Tatsumi et al., 2009).

These clinical results have also been replicated in a mouse model, further contributing to the understanding of the mechanism of action of Hochuekkito (Yae et al., 2012). In this study on the effects of Hochuekkito on colon adenocarcinoma induced cachexia in mice, treatment did not inhibit tumor growth but significantly attenuated the reduction in carcass weight, food and water intake, weight of the gastrocnemius muscle and fat tissue around the testes, and decrease of serum triglyceride level compared with controls. Serum IL-6 level and IL-6 expression level in macrophages in tissues surrounding the tumor was also significantly reduced. In subsequent *in vitro* experiments Hochuekkito suppressed the production of IL-6 by THP-1 or RAW264.7 macrophages. These results suggest that Hochuekkito inhibits the production of proinflammatory cytokines, particularly IL-6, by macrophages within the tumor microenvironment. It may therefore be a promising anticachectic agent for the treatment of patients with cancer.

For the Juzentaihoto “Hozai” prescription, the amount of clinical data that have been documented concerning a use against cachexia is significantly smaller. As a significant correlation between inflammatory processes in the liver and the development of hepatic cellular cancer (HCC) has been demonstrated, chronic inflammation is considered to be one of the prime targets for therapeutic intervention to prevent both the progression of chronic liver disease into cancer and the relapse of HCC (Tsuchiya et al., 2008). Tsuchiya M et al. proposed that Juzentaihoto is protective against hepatocarcinogenesis by hampering Kupffer cell-induced oxidative stress in a clinical study (Tsuchiya et al., 2008). After surgical treatment of HCC, 48 patients were randomly assigned to receive Juzentaihoto or no therapy for up to 6 years. HCC reoccurred in most of the patients, but nevertheless their intrahepatic recurrence-free survival was significantly longer in the Kampo group. These data demonstrate that intrahepatic recurrence-free survival after surgical treatment of hepatocellular carcinoma is improved by Juzentaihoto. In order to investigate the mechanism of action of Juzentaihoto, an aqueous solution of diethylnitrosamine was administered for 22 weeks to male mice that were either fed regular chow or a diet containing Juzentaihoto. In the subsequent experiment, the authors evaluated liver tumor incidence, cell proliferation, and cytokine expression. Juzentaihoto hampered the development of liver tumors in mice and as well as oxidative DNA damage, inflammatory cell infiltration and cytokine expression. We can thus assume that the inhibition of Kupffer cells plays an important part in the protective mechanism of Juzentaihoto, resulting in lower levels of pro-inflammatory cytokines and oxidative stress in the liver. This may in turn slow down the process of hepatocarcinogenesis and improves hepatic recurrence-free survival in patients with HCC. Due to the close correlation between these inflammatory processes and cachexia, one should assume that Juzentaihoto should also be an effective therapy for the latter.

However, presently only an animal study has been published for this indication by Choi et al. (2014) in Korea, where Juzentaihoto is known under the name “Sipjeondaebo-tang”, using a CT-26 tumor-bearing mouse cancer anorexia/cachexia model. In this model, Juzentaihoto was much more effective in the treatment of anorexia and cachexia in a preventive approach. Moreover, Juzentaihoto inhibited the production of IL-6, MCP-1, PYY, and GLP-1 and thus ameliorated cancer-induced anemia. This study therefore also suggests that Juzentaihoto may be useful for treating cancer associated anorexia and cachexia (Choi et al., 2014).

A third traditional Kampo prescription that is often used in the therapy of cachexia is Rikkunshito (六君子湯) (Rikkunshito, 2017). A comparison of its composition with that of Hochuekkito reveals several overlaps—especially Ginseng Radix, Glycyrrhizae Radix, and Zingiberis Rhizoma—the latter of which has already been recommended as a health food for cachexia patients in the West (Azhar and Wei, 2006). Consequently, similar pharmacological effects are to be expected.

It is therefore not surprising that Tomono et al. (2006) have reported that Rikkunshito administered in combination with the anti-emetic drug granisetron alleviated anorexia and vomiting in patients with advanced breast cancer undergoing chemotherapy. Another clinical study found that the median survival of cachectic pancreatic cancer patients treated with gemcitabine was prolonged by co-medication with Rikkunshito (Fujitsuka et al., 2011). This effect was especially notable in patients suffering from ascites. In the same report, Rikkunshito reduced hypothalamic corticotropin-releasing factor (CRF) levels and improved anorexia, gastrointestinal dysmotility, muscle wasting, and anxiety-related behavior in an animal model resulting in a prolonged survival of the test animals. Subsequent *in vitro* experiments suggest that Rikkunshito exerts a sensitizing effect on ghrelin signaling that may be essential for ameliorating anorexia-cachexia and prolonging survival (Fujitsuka et al., 2011).

Especially ghrelin resistance has been linked to the development of cachexia in cancer patients as anorexia is often observed in these patients despite an elevation of ghrelin. In a rat model of cancer cachexia induced by human gastric cancer-derived 85As2 cells (Terawaki et al., 2018), 85As2-tumor-bearing rats developed severe cachexia symptoms, including anorexia and loss of body weight/musculature, whilst responding poorly to intraperitoneal injections of ghrelin (Terawaki et al., 2014). Oral administration of Rikkunshito to these ghrelin resistant rats for 7 days partly alleviated the poor response to ghrelin and ameliorated anorexia without affecting the elevation of plasma ghrelin levels, indicating that Rikkunshito may be a promising agent for the treatment of cancer cachexia (Terawaki et al., 2017).

Even outside of the confines of cancer therapy, Rikkunshito was shown to significantly improve food intake in a clinical study on six elderly dementia patients over four weeks of therapy (Utumi et al., 2011). Other parameters, such as body weight and albumin in plasma, did not change significantly during the examination, although they slightly increased in some patients. For an overview of all the above mentioned human trial data, see **Table 2**.

**Table 2:** Comparison of clinical studies on the use of Kampo preparations in the treatment of cachexia and related forms of wasting syndromes.

Underlying Disease	Kampo prescription	Definition used	Number of Patients (n = x)	Time Frame	Side effects	Outcome	Reference
Genitourinary cancer	Hochuekkito (3 x 2.5 g/d)	Patients complaind about anorexia and lassitude	162	1 - 142 weeks (Ø = 20.1 weeks)	Mild gastrointestinal disorders (n = 12; 7.4%)	63.0% improvement overall 48.4% for anorexia 36.6% for lassitude	Kuroda et al. (1985)
Chronic Fatigue Syndrome (CFS)	Hochuekkito (3 x 2.5 g/d)	Performance Status of 2 or more (CFS diagnostic standard of the Japanese ministry of health) NK activity	29	8 - 12 weeks	Symptoms worsened slightly in 2 cases	41.4% improvement overall	Kuratsune et al. (1997)
Diverse types of cancer	Bojungikki-tang (Jap. Hochuekkito) (3 x 2.5 g/d)	Baseline global fatigue level ≥ 40 mm in the 100-mm Visual Analogue Scale of Global Fatigue (VAS-F)	40 (20 treatment + 20 control)	2 weeks	2 patients reported flatulence and dyspepsia	VAS-F; -1.1 ± 2.1 vs 0.1 ± 0.9, P < 0.05 FACT-G; 3.7 ± 9.9 vs -2.4 ± 9.5, P < 0.05 FACT-F; 8.0 ± 13.6 vs -2.2 ± 14.1, P < 0.05 TOI-F; 6.5 ± 9.2 vs -0.5 ± 10.9, P < 0.05	Jeong et al. (2010)
Old age	Hochuekkito (3 x 2.5 g/d)	Short Form 36 Health Survey (SF-36) Profile of Mood States (POMS) NK activity	13 (active–placebo 4 + placebo–active 5 + active–active 4)	16 weeks (2 weeks run-in + 2 x 2 weeks therapy + 2 weeks washout)	No adverse effects observed	Physical component summary (PCS) of SF-36 improved significantly (p = 0.018) 4 of 6 components of POMS improved significantly	Satoh et al. (2005)
Chronic obstructive pulmonary disease (COPD) Old age	Hochuekkito (3 x 2.5 g/d)	Systemic inflammation Nutritional status	71 (study group 34 + control group 37)	6 months	No adverse effects observed	Body weight increased (from 50.9 ± 2.0 kg to 52.2 ± 1.9 kg) (P < 0.05) and CRP, TNF-α, IL-6 decreased with treatment No change in control	Tatsumi et al. (2009)
Hepatocellular carcinoma (HCC)	Juzentaihoto (3 x 2.5 g/d)	Patients had surgical treatment for liver cancer	48 (Kampo 10 + no supplementation 38)	6 years	No adverse effects observed	Recurrence-free survival time of 49 months with treatment; 24 months in control (P = 0.023)	Tsuchiya et al. (2008)
Pancreatic cancer	Rikkunshito (3 x 2.5 g/d)	Stage III/IV pancreatic cancer with ascites	39 (Gemcitabine 33 + Gemcitabine-Rikkunshito 6)	Till death of all patients	Not reported	Median survival 224 days vs 378.5 days (P < 0.05)	Fujitsuka et al. (2011)
Dementia Old age	Rikkunshito (3 x 2.5 g/d)	Dementia Old age	6	4 weeks	Mild lower limb oedema appeared in 2 patients	Body weight increased (from 41.4 ± 6.5 kg to 43.6 ± 7.0 kg) with treatment (P < 0.10)	Utumi et al. (2011)

In animal models of aging, a connection to the ghrelin signaling pathways has been proposed for these cases of gerontological cachexia as well. It has been hypothesized that ghrelin has a role in protecting against aging-related diseases under caloric restriction. In a series of experiments on Klotho-deficient, SAMP8 and ICR mice as models of aging and GHS-R knockout mice as animal models of accelerated or normal human aging, ghrelin antagonists hastened death whereas ghrelin signaling potentiators like Rikkunshito ameliorated several age-related diseases and prolonged survival in klotho-deficient, SAMP8 and aged ICR mice. Pericarditis, myocardial calcification and atrophy of myocardial and muscle fibers were also improved by treatment with Rikkunshito (Fujitsuka et al., 2016).

Besides the abovementioned, intensively researched “Hozai”, there are several further Kampo prescriptions that are commonly used in the classical “Hozai” indication—e.g. wasting disease and anorexia. Among these, the prescription Ninjinyoeito (**Table 3**) deserves special mention, as it is commonly used—with good clinical evidence—for cachexia therapy. Ninjinyoeito has been recommended for the “improvement of weakening according to chronic disease”, “after operation” and “after radiotherapy” (Yasui, 2005) and is commonly used by Kampo doctors for the prevention and/or treatment of cachexia, frailty and related conditions. Although this prescription has been described for the treatment of these conditions (Amitani et al., 2015), and promising data from *in vitro* experiments have been published (Miyano et al., 2020), clinical studies of statistically sufficient scale are unfortunately missing. However, as an individual case, the successful treatment of tumor cachexia in a patient with lung carcinoma has been reported in clinical literature (Kamei et al., 2000). Ninjinyoeito is therefore currently under consideration for inclusion into the upcoming edition of the Japanese Pharmacopoeia in a couple of years.

**Table 3:** Raw drugs and their respective daily dosages commonly used in the Ninjinyoeito prescription.

人参養栄湯 Ninjinyoeito	
JP Rehmannia Root	4.0 g
JP Japanese Angelica Root	4.0 g
JP Atractylodes Rhizome	4.0 g
JP Poria Sclerotium	4.0 g
JP Ginseng	3.0 g
JP Cinnamon Bark	2.5 g
JP Polygala Root	2.0 g
JP Peony Root	2.0 g
JP Citrus Unshiu Peel	2.0 g
JP Astragalus Root	1.5 g
JP Glycyrrhiza	1.0 g
JP Schisandra Fruit	1.0 g

As far as the dosage of Kampo “Hozai” prescriptions is concerned, in most cases the standardized dosage of 3x2.5 g/d extract granules for most Japanese health insurance-covered Kampo extract-preparations is applied. However, especially in the case of Ninjinyoeito and in the therapy of patients of highly advanced age, a dosage of 2x3.75 g/d extract granules of a finished pharmaceutical product Kampo extract-preparation is increasingly used for the better compliance of the patients.

In contrast to East Asian traditional herbal medicine, well documented therapy options from Traditional European Herbal Medicine are relatively rare. The single exception is the Russian “Adaptogen” concept, which is astonishingly similar to the Japanese “Hozai” concept. “Adaptogens” are defined as nontoxic compounds, revealing polyvalent action and pharmacological effects related to the ability to adapt and to survive. Most importantly, they have normalizing effects on various biomarkers and symptoms of diseases, e.g. the cortisol level in the blood, arterial pressure, gastrointestinal pH in stomach ulcers, etc. Initially, the term “Adaptogen” was coined to describe substances that can increase the “state of non-specific resistance to stress” by Nicolai V. Lazarev (Lazarev, 1958; Lazarev et al., 1959). When the “Adaptogen” concept was first proposed in the 1950s, eight Russian medicinal plants were identified by Lazarev as the “Classical Adaptogens”: *Aralia mandshurica* Rupr. et Maxim., *Echinopanax elatum* Nakai, *Eleutherococcus senticosus* (Rupr. & Maxim.) Maxim., *Leuzea carthamoides* (Willd.) DC., *Panax ginseng* C.A.Mey., *Schisandra chinensis* (Turcz.) Baill., *Rhodiola rosea* L., and *Sterculia platanifolia* L.f. Amongst these, *Panax ginseng* C.A.Mey. is notably a component of all four above discussed Japanese Kampo preparations. In Sweden, Norway and Denmark, a *Rhodiola rosea* traditional herbal medicinal product is indicated as an “Adaptogen” in situations of decreased performance such as fatigue and sensation of weakness.

In this context, it is important to note that *R. rosea* is the only classical “Adaptogen” that has been systematically researched for its activity against cachexia (Chen et al., 2016). The pharmacological effects of *R. rosea* preparations are often largely attributed to salidroside. Here, this phenylpropanoid glycoside was tested in a mouse model of cachexia induced by CT-26 and Lewis lung carcinoma (LLC) tumor, respectively. The main characteristics of cancer-associated cachexia were diagnosed after treatment with salidroside and/or chemotherapy. Western blot was used to determine the levels of several critical muscle-related signal proteins such as mammalian target of rapamycin (mTOR), p-mTOR, and myosin heavy chain (MyHC). In both the CT-26 and LLC models administration of salidroside preserves the tumor-free body weight, decreases the loss of adipose tissue and gastrocnemius muscles, alleviates tumor burden, and prolongs their survival time. The anti-tumor activity of cisplatin was improved through co-medication with chemotherapy, decreasing or even eliminating chemotherapy-induced cachexia. Molecular analysis demonstrated that salidroside significantly increased the expression of mTOR, p-mTOR, and MyHC in gastrocnemius muscle, all of which have been associated with a build-up in muscle mass in the mammalian body. The expression of these factors was not only increased by the substance but was furthermore able to reverse their tumor necrosis factor-α induced down-regulation. In general, the presently accessible experimental data imply that the activity of salidroside—and by extension of *Rhodiola rosea* extracts—on cancer-associated cachexia could be a fruitful approach for a multi-targeted therapy (Chen et al., 2016).

In summary, state of the art clinical, animal model, and *in vitro* research clearly demonstrates the efficacy of the traditional Japanese Kampo “Hozai” prescriptions Hochuekkito, Juzentaihoto, Rikkunshito and also Ninjinyoeito in the therapy of cachexia. Consequently, they are industrially produced in Japan in the form of readymade extract granule preparations of highest quality on an international standard and covered by the National Health Insurance. As there is currently no accepted pharmacotherapy option for cachexia available in the West, a transfer of these Japanese gold standard prescriptions into the European marked would be highly desirable. Herbal medicinal products based in the Russian “Adaptogen” concept do offer an additional therapy option. As there are almost no clinical trials on the use of these drugs in cachexia, more research in this field is urgently needed in order to provide effective treatments for cachexia patients.

## Author Contributions

Both authors researched the literature and wrote the text together.

## Funding

This project was supported by the “Förderkreis der Forschungsstelle für Fernöstliche Medizin”.

## Conflict of Interest

The authors declare that the research was conducted in the absence of any commercial or financial relationships that could be construed as a potential conflict of interest.
